# An fMRI study dissociating distance measures computed by Broca's area in movement processing: clause boundary vs. identity

**DOI:** 10.3389/fpsyg.2015.00654

**Published:** 2015-05-20

**Authors:** Andrea Santi, Angela D. Friederici, Michiru Makuuchi, Yosef Grodzinsky

**Affiliations:** ^1^Department of Linguistics, University College LondonLondon, UK; ^2^Department of Neuropsychology, Max Planck Institute for Human Cognitive and Brain SciencesLeipzig, Germany; ^3^Edmond and Lily Safra Center for Brain Research and Language, Logic and Cognition Center, The Hebrew University of JerusalemJerusalem, Israel; ^4^Institute of Neuroscience and Medicine (INM-1), Forschungszentrum JülichJülich, Germany

**Keywords:** fMRI, working memory, syntactic processing, movement, Broca's area

## Abstract

Behavioral studies of sentence comprehension suggest that processing long-distance dependencies is subject to interference effects when Noun Phrases (NP) similar to the dependency head intervene in the dependency. Neuroimaging studies converge in localizing such effects to Broca's area, showing that activity in Broca's area increases with the number of NP interveners crossed by a moved NP of the same type. To test if NP interference effects are modulated by adding an intervening clause boundary, which should by hypothesis increase the number of successive-cyclic movements, we conducted an fMRI study contrasting NP interveners with clausal (CP) interveners. Our design thus had two components: (I) the number of NP interveners crossed by movement was parametrically modulated; (II) CP-intervention was contrasted with NP-intervention. The number of NP interveners parametrically modulated a cluster straddling left BA44/45 of Broca's area, replicating earlier studies. Adding an intervening clause boundary did not significantly modulate the size of the NP interference effect in Broca's area. Yet, such an interaction effect was observed in the Superior Frontal Gyrus (SFG). Therefore, the involvement of Broca's area in processing syntactic movement is best captured by memory mechanisms affected by a grammatically instantiated type-identity (i.e., NP) intervention.

## Introduction

There is extensive evidence that Broca's area is taxed by sentences with *movement* both from neuropsychological studies of patients and neuroimaging studies of healthy adults (Just et al., 1996; Stromswold et al., 1996; Caplan et al., 1999; Ben-Shachar et al., 2003, 2004; Fiebach et al., 2005; Grewe et al., 2005). Less complex relations, such as simple phrasal composition and local agreement have also been shown to activate/depend on this region (Pallier et al., 2011; Carreiras et al., 2012), however, they have not done so as consistently across methods and populations, as movement (for lack of evidence for simple composition in imaging see Humphries et al., 2005; Brennan et al., 2012). Our goal in this paper is to push our understanding of this special relation between movement and Broca's area even further.

Recent work suggests that activation of Broca's area with syntactic movement may be specifically tied to memory interference, as activity appears to increase with each additional NP intervener within the movement dependency (Santi and Grodzinsky, 2007b; Makuuchi et al., 2013). In the current fMRI study we ask whether this interference effect is modulated by the number of intervening clause boundaries (0 vs. 1). As a clausal boundary increases the number of movements, this manipulation is particularly relevant to theories that place a special role for Broca's area in computing movement dependencies (Grodzinsky, 2000; Grodzinsky and Santi, 2008). Below we elaborate on the structural properties of movement that can be cashed-in as costly for processing mechanisms potentially located within this brain region. While many theoretical positions have been put forth in accounting for this effect, we will argue for the strength of an interference-based account, where interveners are of the same syntactic/semantic type as the moved phrase (type-identical interference henceforth), as opposed to others, for example the number of iterations of a local movement operation.

In sentences with *Movement* (2), a single Noun has (at least) two dependent positions that provide distinct interpretations (e.g., in (2) interrogative and thematic). Only one of these positions is pronounced (2,3), the other(s) copy is <bracketed>, silent and is where the noun is interpreted (thematically) as an argument of a predicate. In contrast, sentences without movement (1) have no silent copy and only one interpretive position for each noun.

1. The boy likes the girl.
2. **Who** does the boy like <***who***>?
3. **Who <*who*>** likes the boy?

Many investigations into movement processing have been based on the object vs. subject movement asymmetry. Object movement (2) unlike subject movement (3) has lexical material intervening between the pronounced position of the noun and where it gets thematically interpreted. Furthermore, the ordering of arguments is non-canonical in the case of object movement (Object-Subject-Verb, above).

The difficulty associated with processing object compared to subject movement has been largely attributed to the degree of referential similarity between the intervening argument(s) and the moved one (Gordon et al., 2001). In a behavioral study, Gordon et al. (2001) studied subject and object extracted relative clauses whereby the head of the relative clause was an NP that was a definite description (e.g., “the barber” in 4 and 5) and the NP within the relative clause was either also descriptive (e.g., “the lawyer”) or a proper name (e.g., “Joe”). Reading times at the two critical words (those underlined in the example sentence in 4 and 5) demonstrated an interaction. Reading times were longer for object-extracted relative clauses compared to subject-extracted ones, when the NP within the relative clause was of the same type as the filler (i.e., descriptive). When a proper name was used there was little if any difference between object and subject extracted relative clauses.

4. The barber that **the lawyer/Joe** admired <*the barber*> climbed the mountain.
5. *The barber* that <*the barber*> admired the *lawyer/Joe climbed* the mountain.

This result demonstrates that the parser is sensitive to the syntactic and/or semantic similarity of features (e.g., +sing, +animate, +definite) between referential items.

Additional behavioral studies have reinforced the idea that long distance dependencies, more generally, are difficult to process when there is a similar intervener. These studies do not focus on referential features, but the syntactic position of the intervening material (Van Dyke, 2007). For example, a subject of a complement clause creates more interference within a subject-verb dependency than does the same NP within an object PP. Thus, a broad range of features (+nom, +animate, +singular, +definite, etc.) may contribute to similarity-based interference during dependency resolution, but their degree of contribution may depend on the particular dependency under investigation.

The finding that Broca's area is sensitive to object movement (Just et al., 1996; Stromswold et al., 1996; Caplan et al., 1999; Fiebach et al., 2005; Grewe et al., 2005) is reinforced by more sophisticated parametric fMRI studies. These studies quantified how taxing movement is by the amount of intervening units between the dependent elements, where units (i.e., “interveners”) have most often been defined as animate, singular, descriptive NPs. Animate, singular descriptive NPs were selected, as they share syntactic and semantic features with the moved phrase, thereby introducing semantic/syntactic identity based interference in memory processes (Gordon et al., 2001). For an example of a parametric manipulation of number of similar interveners, see 6(a–d) from Makuuchi et al. (2013).

6. *a. Ich glaube, der Mann zeigte dem Kind den Onkel gestern Abend.*
b. I think, the_NOM_ man showed the_DAT_ boy the_ACC_ uncle last evening.
c. I think, *the_DAT_ boy* **the**_NOM_ **man** showed <*the_DAT_ boy*> the_ACC_ uncle last evening.
d. I think, *the_ACC_ uncle* **the**_NOM_ **man** showed **the**_DAT_ **boy** <*the_ACC_ uncle*> last evening.

The baseline sentence is presented in 6a in German and 6b presents the English gloss. In this baseline sentence all arguments are in their base position. In 6c, the direct object has moved in front of the subject (crossing 1 NP) whereas in 6d the indirect object has moved in front of the subject (crossing 2 NPs). Previous parametric studies investigated the neural reflections of the number of NPs crossed (i.e., interveners) by a *single* moved NP (Santi and Grodzinsky, 2007b; Makuuchi et al., 2013) *or* of the number of NPs displaced by syntactic movement (Friederici et al., 2006) across different languages (English, German) and movement constructions (Scrambling, Topicalization, Relative Clauses). Their results provide a neurocognitive generalization: Broca's area is sensitive to movement distance measured by the number of similar interveners (in this case type-identical NPs) that moved NPs cross. In conjunction with the results from additional fMRI studies, this interference appears to be occurring proactively rather than retroactively, given that dependencies, which are not predictable until the tail of the dependency (e.g., reflexive binding and parasitic gaps), do not engage Broca's area (Santi and Grodzinsky, 2007a,b). Thus, it would seem that object movement is taxing due to maintenance of a prediction (i.e., gap for an NP) that crosses type-identical interveners (i.e., NP).

A recent fMRI study (Glaser et al., 2013) showed similarity of an NP intervener to the head of the dependency is critical in driving activation in BA44 and 45 (i.e., Broca's area). This particular study did not assess interference within a movement dependency, but a subject-verb (agreement) dependency. The high interference condition had an intervening subject NP (visitor)^1^ within a complement clause (8), whereas the low interference condition had an intervening NP (that was not subject) within a PP (7). The greater activation within Broca's area for (8) than (7) was interpreted to reflect the main verb (i.e., was complaining) cueing for the retrieval of a subject NP whereby an intervening subject NP resulted in greater interference. Thus, unlike our conclusions above, they assume that similarity-based interference effects in Broca's area occur during a cue-based retrieval.

7. *The client* who had arrived after the important **visitor** that day *was complaining* about the investigation.
8. *The client* who implied that the **visitor** was important that day *was complaining* about the investigation.

Whether interference is occurring proactively or retroactively, conflict resolution can apply in recovering the correct representation (Thompson-Schill et al., 1997; Novick et al., 2005; Thothathiri et al., 2012). Thothathiri et al. (2012) specifically suggest that non-canonical structures activate Broca's area due to syntactic competition between an agent-first hypothesis and the actual syntactic representation, which is patient-first in the cases of object-relatives and passives. Conflict resolution is relied-on to distinguish the correct from the incorrect representation. Thus, conflict resolution may apply following interference and be the basis of the observed activation in Broca's area.

Although there is indication that Broca's area is engaged by interference generated by the number of NP interveners (whether affecting proactive, retroactive, or both aspects of processing) crossed by a movement dependency, movement may engage additional processing mechanisms within this region. However, the nature of the tests conducted thus far cannot address this. Multiple distinct computations within Broca's area is not unreasonable, given that it contains multiple anatomical subregions with presumably distinct functions (Amunts et al., 2010). Our goal in this study is to determine whether movement has effects in Broca's area above and beyond those imposed by the semantic/syntactic identity of intervening NPs within a movement dependency. Specifically, does the number of movements affect activation in any subregions of Broca's area or surrounding regions, as another neurolinguistic account of Broca's area has proposed it is involved in computing syntactic movement (Grodzinsky, 2000). We investigated this with sentences involving iterations of a movement operation (i.e., successive cyclic movement) as compared to sentences with a single movement (within a clause) but with an equal number of NPs crossed by that movement. The following provides a brief description of how movement proceeds successive-cyclically after which we will further elaborate on the complexity dimensions tested.

As discussed above, movement involves an interpretation of a phrase in a position that is not pronounced (i.e., silent copy). In those examples we were concerned with a single clause. By comparison, in sentences with multiple clauses, the wh-phrase (i.e., who) moves from a thematic (i.e., *doer* or *doee*), silent position which it “vacates” (gap) to a “filled” position (filler), in which it is pronounced, by stopping off at the left edge of *each* intervening clause and leaving behind a silent copy in each of them (10). Evidence that the wh-phrase moves through intermediate CPs (i.e., CP3 in 9) on the way to its final destination (i.e., CP2 in 10) comes from grammaticality contrasts, as in (9) vs (10). Both (9) and (10) are composed of 3 clauses (CPs). Note that in (9) the *wh*-phrase (i.e., who) crosses more words than in (10) along the path from thematic interpretation to its pronounced position, but (10) is ungrammatical and (9) is not. This grammaticality contrast can be explained by considering that in (9) the *wh*-phrase has an intermediate landing position available (left edge of CP3) that is not available in (10) because the intermediate position is already filled by another wh-phrase (*which boy*). It has thus been proposed that wh-phrases *must* move successively through each CP on the way to their final landing position, leaving traces or silent copies (identified by phrases in angled brackets) in these intermediate positions, because failure to do so results in ungrammaticality (10). This captures the successive-cyclic nature of *movement*.

9. [_CP1_ I know [_CP2_ *who* the teacher from Norway thinks [_CP3_ <*who*> the boy likes <*who*>]]].
10. 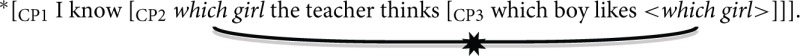

Further, evidence for intermediate landing positions is provided by language acquisition studies, which show children produce wh-words in these intermediate positions (Thornton, 1995). Likewise, Psycholinguistic studies have provided support for intermediate positions (Gibson and Warren, 2004), through demonstrating that intermediate positions ease processing of a sentence-final silent copy relative to comparable length dependencies not involving embedded CPs, achieved through nominalization.

The current study had two design features: (1) we manipulated the number of NP interveners crossed by a moved NP (Baseline:NP/CPS0, 1NP intervener:NP/CP/O1, 2NP intervener:NP/CPO2 in Table 1) and (2) compared successive cyclic movement to a single movement while controlling for number of intervening NPs (see Table 1). The first part of the design allowed us to relate the novel design/results to previous results that investigated a parametric manipulation in the number of intervening NPs. The second part allows us to test whether number of movements has an effect above the number of similar NPs crossed.

**Table 1:** **Example Stimuli**.

**CP INTERVENER SENTENCES**	
CPS0	I said the neurosurgeon knew *which resident* liked the porter
CPO1	I said the neurosurgeon knew [*which porter* **the resident liked** <which porter>]_CP1_
CPO2	I knew [*which porter* **the neurosurgeon said**]_CP2_ [<which porter> **the resident liked** <which porter>]_CP1_
**NP INTERVENER SENTENCES**	
NPS0	I knew *which neurosurgeon* showed the resident to the porter
NPO1	I knew *which resident* [**the neurosurgeon**]_NP1_ showed <which resident> to the porter
NPO2	I knew *which porter* [**the neurosurgeon**]_NP1_showed [**the resident**]_NP2_ to <which porter>

The CP conditions include CPS0, CPO1, CPO2, and the NP conditions include NPS0, NPO1, NPO2. Subject movement conditions that cross 0 NPs (S0) conditions have an embedded wh-subject phrase that does not leave its clause. The object movement condition have an embedded wh-object phrase that crosses one (O1) or two (O2) interveners (defined as either CPs or NPs), respectively. CPS0 and NPS0 along with CPO1 and NPO1 are no different in terms of intervention across a movement dependency.

The baseline condition (CP/NPS0) involves a local subject (S) movement, hence crossing 0 similar NPs. This was compared to movement that crossed 1 similar NP (CP/NP O1); in order to accomplish this the object of the most embedded clause was moved across the subject of that same clause. Furthermore, this was compared to movement that crossed 2 similar NPs (CP/NP O2), which was accomplished again via object movement, either across the two subjects of the two most embedded clauses (CP condition) or across the direct object and subject in a single clause, containing a bi transitive verb (NP condition). This contrast of size to similarity addresses what form of information increases complexity of memory mechanisms in Broca's area. Thus, by comparing condition CPO2 to CPO1 we have a contrast in number of CPs crossed (2 vs. 1) and in contrasting NPO2 to NPO1 we have a contrast in number of NPs crossed (2 vs. 1). Furthermore, collapsing across the two types of interveners we can re-assess the parametric effect of number of similar interveners in comparing the current work to past results.

Although our primary interest was in investigating the effect of multiple movements, it is important to note that multiple movements have a couple of consequences that in and of themselves may increase processing complexity. The multiple movements coincide with a larger syntactic size of the “interveners” (i.e., CP) or put otherwise refers to movement that crosses a clausal boundary. In successive-cyclic movement we are crossing multiple clauses rather than a single one containing some multiple of NPs. CPs contain many more functional projections (i.e., CPs, and tense and agreement checking nodes) and as such are syntactically more complex^2^. Wagers and Phillips (2014) show that movement within a clause involves active maintenance of both coarse (e.g., category) and fine-grained (lexical semantic) information about the antecedent, but across clauses there is active maintenance of just the coarse-grained information, whereby fine-grained lexical information needs to be retrieved at the gap. Thus, a clause boundary manipulation should engage retrieval processes more than one without.

We can test whether crossing a clausal boundary of a wh-movement dependency has an effect on the fMRI signal above that of similarity of the intervener (i.e., NP) to the moved constituent by comparing crossing of 2CPs to 1CP with crossing 2NPs to 1NP (in a single clause). Note, in Table 1, the type contrast (CP, NP) does not differ in terms of dependency distance when there is either 1 or 0 intervener. Thus, one would only expect a difference between the intervener types when comparing 2 vs. 1 intervener (i.e., hence CPS0 is grayed out in Table 1 to highlight the conditions contributing to the expected interaction effect). In summary, an enhancement of activation for crossing a clause could indicate 1 of 2 related processes: (1) number of movements or (2) taxing retrieval mechanisms more due to crossing a clausal boundary.

Any results from the current study that demonstrate an effect of number of CPs over NPs cannot distinguish between number of movements, syntactic size of the intervening material and crossing a clausal boundary. Nonetheless, the data will critically show whether or not Broca's area is sensitive to movement (and syntactic size or crossing clausal boundary) beyond similarity of the interveners to the head of the dependency. The potential complexity factors induced by successive-cyclic movement above type-identical based interference are interrelated (perhaps, reflections of different levels of analyses) and as such not easily disentangled, these include: (1) number of movements (and silent copies) (2) syntactic size of intervening material (between the pronounced and thematically interpreted NP), which involves the crossing of a clausal boundary.

## Methods

### Subjects

Twenty one subjects participated in the study (after exclusion of two participants from the analysis due to low behavioral performance in the fMRI study (<65%)^3^. The average age of participants was 19.90 years, and 12 were female. All subjects were right-handed according to the Edinburgh Handedness Inventory (Oldfield, 1971), had normal or corrected-to-normal vision, a score above 3 on the Daneman and Carpenter Reading Span Test (Daneman and Carpenter, 1980), and gave informed consent in accordance with the ethics committee of the Montreal Neurological Institute (MNI).

### Stimuli

The design of the stimuli crossed NUMBER of intervener (0, 1, 2) with TYPE of intervener (CP, NP). Although as pointed out in the Introduction, the distinction across “Type” for our purposes only arises when the Number of interveners is 2. Each condition was made up of 40 sentences and every sentence was between 17 and 19 syllables in length. In the Intervening CP condition there were two embedded clauses allowing for two successive-cyclic movements from baseline. In the intervening NP condition, to allow for movement over multiple NPs, but not CPs, there was one embedded clause that contained a double-object verb. The following sections contain further detailed descriptions of the parameterization of distance for each intervener type (see Table 1 for example stimuli and Supplementary Materials for full list of Stimuli).

#### Intervening CPs

All sentences started with a pronoun (I, we, he, she) followed by a verb that takes a sentential complement (thought, claimed, hoped, said). The sentential complement was composed of an NP and another verb (knew, learned, announced) that takes a sentential complement. This second embedded clause was composed of an NP, a verb and direct object NP. In CPS0 there is no movement over a clause but there is (or may be) movement into a CP (unless one does not assume string vacuous movement)^4^. From baseline there is movement of the second embedded object to the front of the second embedded clause (CPO1 in Table 1), or movement of the second embedded object to the front of the first embedded clause (CPO2).

#### Intervening NPs

Likewise in the NP condition the sentences began with a pronoun (I, we, he, she) followed by a verb that takes a sentential complement (knew, announced, learn), the sentential complement was made up of a subject NP, a double object verb (introduced, described, showed, recommend) and its direct object NP and indirect object NP. In the baseline condition, NPS0, there is no movement over an NP, but there is (or may be) movement of the subject into a CP (unless one does not assume string vacuous movement). From baseline there is movement of the direct object in front of the embedded subject (NPO1). The second parameterization moved the indirect object over both the direct object and embedded subject (NPO2).

### Procedure

To assure the participants were processing and understanding the sentences a yes/no question about stimulus content followed 50% of the sentences. Half of these required a “yes” response and half a “no” response. Questions requiring a “no” response involved a thematic role reversal (see 7–8 below). Given the difficulty of the task, we wanted to be assured that there would be a low exclusion rate in the fMRI study. Thus we screened subjects before fMRI scanning for behavioral performance days to weeks before the actual fMRI session. During screening, participants performed the task on 50% of the stimuli and were included for the fMRI study if they performed at 75% or greater in every condition. We screened 52 people whereby 28 satisfied all requirements (including handedness, language and behavioral performance). Of the remaining 24 that did not satisfy the screening requirements, 14 of them did not satisfy the requirements for behavioral performance alone. Of those 14, 10 still scored above 75% on average across all conditions. Thus, many simply performed below the conservative threshold on 1 or 2 of the conditions. Half of the subjects were screened on one-half of the sentences and the other on the complementary set. Both groups of participants saw the complementary set of comprehension questions from their screening session in the actual fMRI study, and both saw the entire set of sentences (the full set of items across all conditions). Thus, the half they saw in practice they saw again during the fMRI study that was run days or weeks later. Therefore, comprehension sentences only appeared on 50% of the trials in the fMRI study.

7. I said the neurosurgeon knew which porter the resident liked.
8. Did I say the neurosurgeon knew which resident the porter liked?

**Figure 1:**
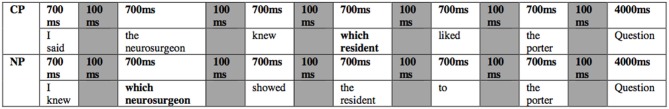
**Trial Dynamics of sentence presentation phrase/word by phrase/word**.

The stimuli were programmed with Presentation software (Neurobehavioral Systems, Inc., Albany, California, USA) on a Windows PC. The stimuli were projected onto a screen at the back of the MRI and then reflected into a mirror attached to the head coil. The sentences appeared word/phrase by word/phrase (see Figure 1). Each word/phrase appeared for 700 ms with 100 ms between. The comprehension question was presented for 4000, 100 ms after the sentence. On trials without comprehension questions there were 3 scans (4.8 s) of blank screen inter-trial interval (ITI), whereas on trials followed by comprehension questions there were 2.5 scans (4 s) of ITI. Half of the stimuli were presented in each of two runs. See Figure 1 for a depiction of the trial dynamics. Trial order and additional interspersed silence (10^*^12.8 + 10^*^9.6 s) for jittering stimulus onset was optimized by optseq (http://surfer.nmr.mgh.harvard.edu/optseq/) with the presentation of the trial being jittered by 0 or 800 ms from the onset of the scan. Run order was counterbalanced across participants. An MRI compatible response box for comprehension question responses was placed in the participants' left hand to avoid potential motor activation overlapping with typically left frontal language activation.

### Image acquisition

Functional and structural data were acquired on a 3T Siemens magnetom Triotim. Twenty-six slices, 4 mm thick oriented AC-PC, with full coverage of the frontal, temporal, and occipital lobes and partial coverage of the parietal lobes were acquired (*TR* = 1.6 s, *TE* = 30 ms, Flip angle = 90°, FOV = 25.6 × 25.6 cm^2^, 64 × 64 matrix). Superior aspects of the parietal lobe could not be included to maintain the desired functional and anatomical resolution. Voxels were 4 × 4 ×4 mm in volume. There were 176, 1 mm thick structural scans acquired with an MPRage sequence (*TR* = 2300 ms, *TE* = 2.98 ms, FOV = 256 × 240 mm, 256 × 240 matrix). During scanning, an air vacuum pillow and sponges were used to stabilize the head.

### Analysis

#### Behavioral data

Mean reaction times (RT) and accuracy for each subject and condition was entered into a 2 TYPE (NP, CP) by 3 Distance (0, 1, 2) Repeated Measures ANOVA (both by subjects and by items).

#### fMRI data

The first 4 volumes of each fMRI run were removed from the analysis, in order to exclude magnetic saturation effects. The data were analyzed in SPM8 (available at http://www.fil.ion.ucl.ac.uk/spm/). Functional images were aligned to the first image and resliced in order to correct for motion. Then coregistration between functional and anatomical images was performed. Anatomical images were segmented and normalized to MNI space. The resultant transformation matrix was applied to the functional images that were subsequently spatially smoothed with an 8 mm FWHM Gaussian kernel. The data were modeled with regressors for each sentence condition and 1 regressor for all comprehension questions and convolved with a canonical model with a time derivative. The time derivative was applied to handle slice timing differences (Henson et al., 1999). A high pass filter with a cut-off of 128 s was applied to the data. The contrast images for each condition of each subject were submitted to a second-level (group) analysis: (1) 2TYPE(CP, NP) × 3Number(S0, O1, O2) within-subject ANOVA. *F*-test of the interaction was FWE corrected for multiple comparisons. *T*-tests were used to test for a linear effect of Number [-1 0 1 -1 0 1] (CP/NPO2>CP/NPO1>CP/NOS0) to replicate previous studies that have demonstrated an effect of number of NPs intervening a movement dependency. Main effects of Type (CP>NP) and (NP>CP) were coded as *t*-tests as well [1 1 1 -1 -1 -1] and [-1 -1 -1 1 1 1], respectively. These effects compare multiple syntactic factors (e.g., verb argument structure, number of clauses) so the interpretation of any such results need to be made with caution, but nonetheless provide further data considering syntactic differences in processing. Additionally, the interaction to test for an effect of syntactic size (CPO2-CPO1>NPO2-NPO1) was coded as a *t*-tests [0 -1 1 0 1 -1]. Again, this particular interaction test was to address whether an intervening clause modulates the effect of an intervening NP. The effect of an additional clause vs. NP is only provided by the 2 intervener condition (CPO2 vs. NPO2 condition). The *t*-test maps were thresholded at voxel-wise *p* < 0.005 for signal intensity and by a cluster size where only significant clusters (*p* < 0.05) were reported.

The anatomy toolbox (www.fz-juelich.de/ime/spm_anatomy_toolbox; Eickhoff et al., 2005) was used for the identification of cytoarchitectonic probability of cluster localization. The Marsbar toolbox (Available at http://marsbar.sourceforge.net) was used for extracting Percent Signal Change from clusters.

## Results

### Behavioral results

**Figure 2:**
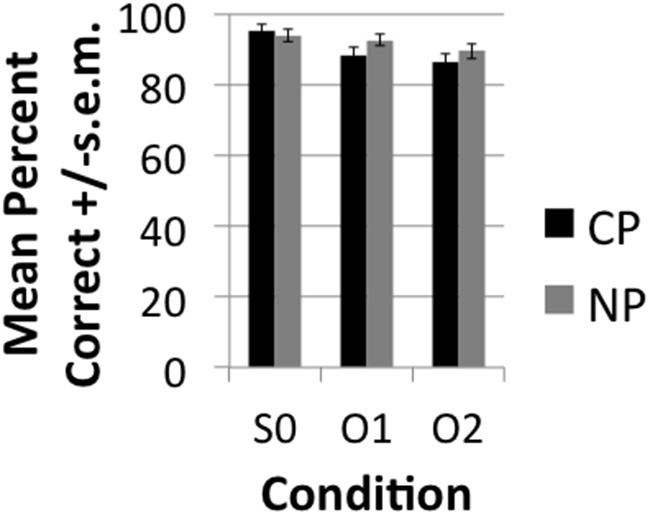
**Mean percent +/−s.e.m. correctly answered comprehension questions broken down by condition**.

**Figure 3:**
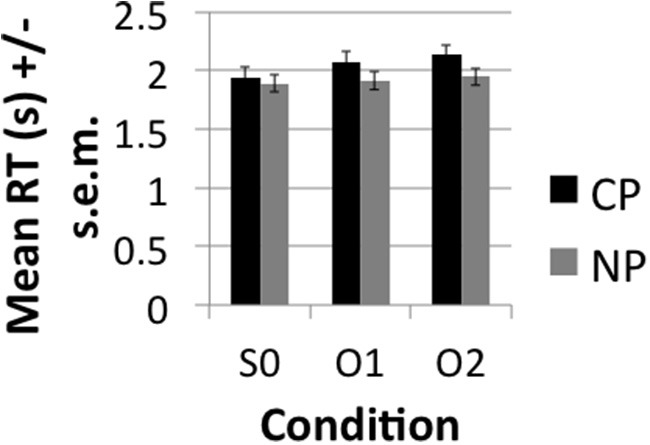
**Mean Reaction Time (RT) in seconds (s) ± s.e.m. to comprehension question broken down by condition**.

The accuracy results demonstrated very high (>85%) accuracy rates (see Figure 2). A main effect of DISTANCE was nevertheless observed over subjects [*F*_1(2, 40)_ = 15, *p* < 0.001] and items [*F*_2(1.476, 57.55)_ = 8.95, *p* < 0.001]. Pairwise comparisons with a Sidak correction for multiple corrections showed that S0 was significantly more accurate than O1 (*p* = 0.007) and O2 (*p* < 0.001), but that O1 and O2 did not significantly differ from one another (*p* = 0.251) both in the subjects and items analysis. Although the conditions did not directly differ there was a significant linear decrease in accuracy (i.e., Linear effect) with DISTANCE in the subjects [*F*_1(1, 20)_ = 37.60, *p* < 0.001] and items [*F*_2(1, 39)_ = 17.19, *p* < 0.001]. This indicates that accuracy demonstrated a decreasing trend with increasing distance even though direct contrasts did not turn out significant. Neither the main effect of TYPE or the interaction of TYPE^*^DISTANCE were significant. The RT results (see Figure 3) likewise demonstrated a main effect of DISTANCE in the subjects [*F*_1(2, 40)_ = 6.94, *p* < 0.003] and items [*F*_2(2, 78)_ = 6.35, *p* = 0.003] and Linear Effect of DISTANCE in the subjects [*F*_1(1, 20)_ = 10.66, *p* < 0.004] and items [*F*_2(1, 39)_ = 12.62, *p* = 0.001]. The main effect of DISTANCE was due to a faster reaction time for S0 than O2 (*p* = 0.012) in the subjects analysis and due to a faster reaction for S0 than both O1 (*p* = 0.03) and O2 (*p* = 0.001). There was also a main effect of TYPE in the subjects [*F*_1(1, 20)_ = 19.23, *p* < 0.001] and items [*F*_2(1, 39)_ = 28.9] analyses and an interaction between TYPE and DISTANCE that was approaching significance in the subjects [*F*_1(2, 40)_ = 3.22, *p* < 0.051], but not the items [*F*_2(2, 78)_ = 1.85, *p* = 0.164] analysis. The trend of an interaction was due to CP interveners having a greater effect on slowing RT with increasing number of interveners than NP interveners. The main effect of TYPE was due to a slower RT for CP (mean = 2.05, SE = 0.084) than for NP (mean = 1.92, SE = 0.073).

### fMRI results

The current study tested whether an additional clause boundary within a wh-movement dependency has an effect on the fMRI signal above that of similarity of the intervener (i.e., NP) to the moved constituent. That is, it tested whether an additional clause boundary would have a greater effect on the fMRI signal than that of NP interveners.

#### Number of intervener NPs

**Figure 4:**
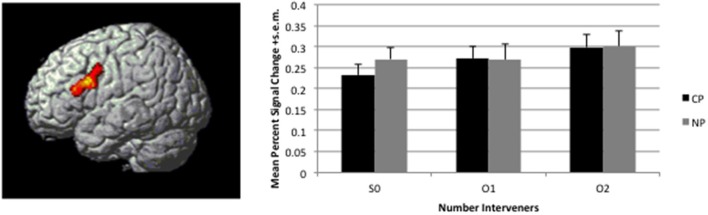
**Linear effect of distance (S0<O1<O2) observed in the 2Type(CP, NP) × 3Number(S0, O1, O2)**. Map is thresholded at voxel-wise *p* < 0.005 and corrected for multiple comparison through cluster size *p* < 0.05. Activation is overlaid on a rendered surface of the brain. The histogram to the right presents the mean percent signal change +s.e.m. over the entire cluster broken down by condition (percent signal change was calculated in Marsbar per subject).

**Figure 5:**
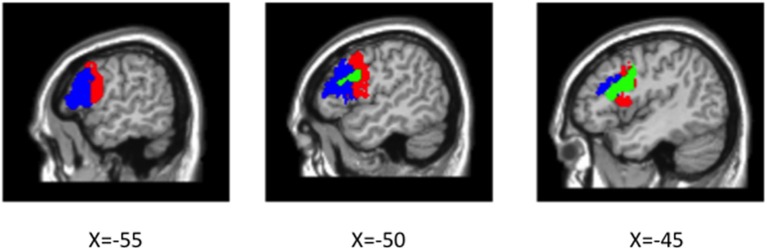
**Cytoarchitectonic probability maps of BA 44 (red) and BA 45 (blue) thresholded at 30% overlap overlaid on canonical average brain and linear main effect (voxel-wise** ***p*** **< 0.005, cluster-level** ***p*** **< 0.05; green) overlaid on top**.

A significant linear effect of Number of interveners was observed bilaterally in the Inferior Frontal Gyrus (IFG) and in the Caudate Nucleus (see Table 2 and Figures 4, 5. for details). The anatomy toolbox, identified the peak LIFG activation (−40, 12 26) was within BA 44 with a probability of 30%. As can be seen in Figure 5, the LIFG activation is strongest in BA44 and spreads into the posterior portion of BA45. Across both the BA44 and 45 probability maps, thresholded at 30% (as in Figure 5), 404 voxels of the linear activation cluster are within the maps (i.e., 50% of the cluster overlaps with the maps). When using unthresholded probability maps (i.e., 10–100%), 590 voxels of the cluster are contained within the probability maps of BA44/45 (i.e., 73%). The activation that is not overlapping with the probability maps is mostly due to medial and posterior extension of the activation. In addition to LIFG activation, both the caudate and right Broca's area demonstrated activation, but this activation occupied a much smaller cluster (about half the size) then that on the left. Further, in fMRI it is difficult to know the necessity of the area(s) activated and from additional studies, it would appear that right Broca's area is often activated (amongst patients and healthy participants), but unlike LIFG, is not causally involved in language processes (Thiel et al., 2006).

**Table 2:** **Regions activated by a linear effect of Distance**.

**Location**	**BA**	**Voxels**	**Clusterp**	**PeakZ**	**Coordinates**
Left Inferior Frontal Gyrus	44/45	812	0.01	4.07	−40 12 26
				3.63	−38 4 34
				3.14	−42 22 16
Caudate Nucleus	–	488	0.038	3.70	−8 10 4
				3.51	8 8 0
Right Inferior Frontal Gyrus	44/45	436	0.048	3.38	52 12 26
				3.24	36 10 26
				2.91	44 4 34

Thresholded at a voxel-wise p < 0.005 and corrected for multiple comparisons by a cluster level p < 0.05.

#### Additional clause boundary

There was no interaction effect in Broca's area, either defined by a linear increase (0 to 1 to 2) that is greater for CP interveners than NP interveners or in terms of the (2–1 intervener) subtraction having a greater effect for CP compared to NP interveners. In the *t*-tests, a significant interaction effect defined by a greater effect of number of interveners (2 vs. 1) in the CP condition than the NP condition was, however, localized bilaterally in the Superior Frontal Gyrus (SFG; see Table 3 and Supplementary Figure).

**Table 3:** **Regions activated by a 2Type(CP, NP) × 2Distance(O1, O2) interaction where (CPO2-CPO1)>(NPO2-NPO1)**.

**Location**	**BA**	**Voxels**	**Clusterp**	**PeakZ**	**Coordinates**
Superior Frontal Sulcus/Gyrus (SFS/SFG)		1696	0.001	4.95	44 38 32
				4.20	−6 42 50
				4.18	40 36 40

Thresholded at a voxel-wise p < 0.005 and corrected for multiple comparisons by a cluster level p < 0.05. This is one cluster that contains the bilateral SFS/G.

#### Effect of syntactic type

**Figure 6:**
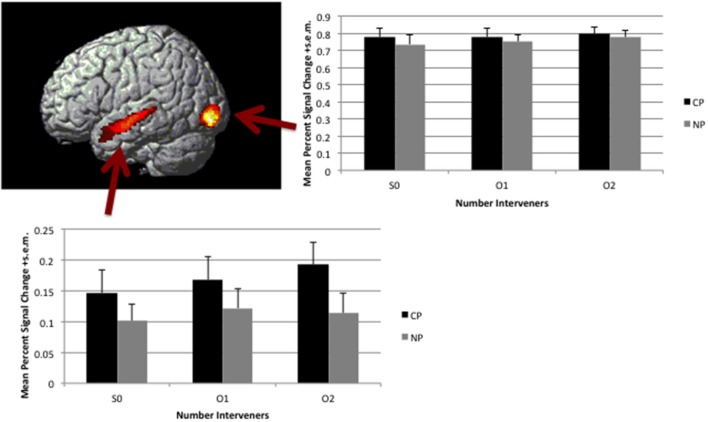
**Main effect of Type (CP>NP)**. Map is thresholded at voxel-wise *p* < 0.005 and corrected for multiple comparison through cluster size *p* < 0.05. Activation is overlaid on a rendered surface of the brain. The histogram to the right presents the mean percent signal change +s.e.m. over the entire Inferior Occipital Gyrus cluster broken down by condition (percent signal change was calculated in Marsbar per subject). The histogram below presents the mean percent signal change +s.e.m. over the entire Superior Temporal Sulcus cluster broken down by condition (percent signal change was calculated in Marsbar per subject).

There was a significant effect of TYPE in the Superior Temporal Sulcus (STS) and the Inferior Occipital Gyrus (IOG, see Table 4; Figure 6). This effect was due to the CP condition producing greater activation than the NP condition. There were no significant clusters that demonstrated greater activation for the NP condition over the CP condition.

**Table 4:** **Regions activated by a main effect of Type (CP vs. NP)**.

**Location**	**BA**	**Voxels**	**Clusterp**	**PeakZ**	**Coordinates**
Left Inferior Occipital Gyrus		611	0.022	5.25	−22 −92 −8
Left Superior Temporal Sulcus		597	0.023	3.91	−56 −8 −12
				3.54	−52 −24 −4
				3.32	−50 −32 0

Thresholded at a voxel-wise p < 0.005 and corrected for multiple comparisons by a cluster level p < 0.05.

## Discussion

The novel result that this study presents is that while Broca's area is sensitive to the number of type-identical interveners in long distance wh-movement, this effect is not augmented by **a clausal boundary**. Other less prominent areas of activation that demonstrated this same effect were found in the right homolog of Broca's area and the caudate nucleus. On the other hand, more superior areas (i.e., SFG) were augmented by a clausal boundary (or the syntactic size of the intervener).

The result in Broca's area is consistent with psycholinguistic data that has demonstrated that the similarity of the interveners to the head of a movement dependency increases processing difficulty (Gordon et al., 2001). Our results further expand on these results in two ways: (1) by demonstrating that a syntactically similar intervener, but not an intervening clausal boundary, increases activation in Broca's area, its right homolog, and the basal ganglia, (2) a clausal boundary further increases complexity of a movement dependency, as evidenced by a marginally significant behavioral effect on offline RTs to verification questions and increased activation within the SFG and to some degree the left superior temporal cortex.

### Broca's area and syntactic/semantic similarity based interference

Broca's area has been repeatedly reported to be engaged by object movement dependencies (Just et al., 1996; Stromswold et al., 1996; Caplan et al., 1999; Ben-Shachar et al., 2003, 2004; Fiebach et al., 2005; Grewe et al., 2005). Here we have explicitly framed movement distance in terms of number of type-identical interveners (NPs). This definition of distance not only holds of our results but also covers some other closely related studies (Santi and Grodzinsky, 2007b; Makuuchi et al., 2013). The cross-study consistency with Makuuchi et al. (2013) holds in terms of anatomical location but also in terms of experimental paradigm (reading word/phrase by word/phrase; comprehension questions assessing thematic role reversal) and methods for data analysis. The two studies differ in terms of the syntactic constructions tested. Makuuchi et al. (2013) investigated two types of movement dependencies in German, Scrambling and Topicalization. Here we studied embedded wh-movement. Similar to what is reported here, Makuuchi et al. (2013) found a linear effect of number of NP interveners on the fMRI signal with a peak in BA44, spreading into BA45.

A previous study by Santi and Grodzinsky (2007b) found the activation for increasing number of NPs within a movement dependency to be centered more anteriorly than the current study. This previous study differed from the current one in many ways. For one, intermittent scanning was used (thus the point of the scan may have been biased to BA45 processing, if BA45 has an earlier or later peak in processing relative to BA44), presentation modality was auditory, and the data analysis was slightly different (parametric effect was taken into consideration in the model). Nonetheless, these three studies are relatively similar and provide corroborating evidence for the role of Broca's area in being sensitive to movement distance defined over intervening constituents (i.e., NPs) that are similar to the head of the dependency.

Based on previous fMRI studies that find *unpredictable* syntactic dependencies (i.e., Reflexive Binding) do not activate Broca's area when NPs intervene, we suggest that the observed similarity-based interference effects are based on predictive processes. In particular, that there is storage of a prediction (NP gap) that is affected by similar, intervening NPs. The implication is that maintaining syntactic predictions increases activation in Broca's area and these predictions are affected by interveners that are identical in type. In the case of a movement relation, the parser is predicting a gap, which could involve storage of a category (i.e., NP) (Wagers and Phillips, 2014) or possibly an even more detailed feature profile (+sing, +animate, +nominal). When a *potential* gap site is reached this may cause reactivation of the entire lexical content of the filler or not (if maintained), but in either case the presence of another (type-identical) NP at a potential gap location will cause interference.

The behavioral data similarly shows that the number of intervening NPs affects both accuracy and RT. However, there is some indication that RT is primarily affected by CPs and not NPs (at least in the analysis by subjects but not the analysis by items). It is important to bear in mind that these are offline measures so how they directly relate to online measures of interference is more difficult to ascertain.

Our conclusions are consistent with Glaser et al. (2013) in showing that Broca's area is activated when there is interference by type-identical NP interveners in resolving syntactic dependencies. However, our conclusions differ in that Glaser et al. (2013) attribute this interference to occur during cue-based retrieval rather than along the prediction path. Remember that Glaser et al. (2013) do not investigate a movement dependency, but nonetheless one that is predictable, a subject-verb dependency (Van Dyke and McElree, 2006; Van Dyke, 2007). Trying to generalize across these two types of dependencies may not be the right approach. Further study is required to establish the degree of similarity in the memory mechanisms used to resolve these two dependencies and whether they are dependent on cue-based retrieval or maintenance of a prediction.

In general, the perspective that Broca's region engages in conflict resolution (Thompson-Schill et al., 1997; Novick et al., 2005; Thothathiri et al., 2012) is compatible with either interference during prediction or cue-based retrieval. In terms of prediction, it would attribute a conflict to wanting to release the filler as soon as possible and the actual representation, in which the “potential” gap site is already filled with a type identical NP. In resolving this conflict, the parser will need to maintain the gap prediction and do so for the correct NP. In terms of cue-based retrieval, this perspective would attribute conflict resolution to deciding between which of the type-identical NPs is the actual argument (head) of the verb or gap.

Lastly, it is worth noting that although about three-quarters of the activation lies within Broca's area (BA44/45), it also extends medially and posteriorly from Broca's area, including areas that connect Broca's area to other regions.

### A syntactic working memory

Given that the syntactic complexity effects observed in Broca's area depend on long distance dependencies, some have argued that its functional role is to provide a syntactic working memory (Caplan and Waters, 1999). The results from this study indicate that, if so, the size of the intervening structure is not as relevant as its similarity to the moved constituent. It appears that the critical dimension is the similarity of syntactic structure/features between the moved phrase and those intervening along the path of movement.

### Phonological working memory

Some would argue that this linear effect of distance in Broca's are is related to a phonological working memory (Rogalsky et al., 2008) rather than syntactic/semantic similarity. However, contradictory evidence has been provided from various other studies (Caplan et al., 2000; Santi and Grodzinsky, 2007b). Even though Rogalsky and Hickok (2009) interpret their results to be due to phonological working memory, even their results demonstrate that there is activation in Broca's area during concurrent speech articulation. Thus, there is no clear evidence indicating that the observed syntactic complexity effect can be reduced to a phonological working memory.

### Basal ganglia, WM, and syntactic complexity

In addition to Broca's area (and its right homolog), a linear effect of interveners was observed in the basal ganglia. Makuuchi et al. (2013) also observed a linear effect of interveners within a movement dependency in the basal ganglia, however, there the activation was observed in the globus pallidus rather than the caudate nucleus. Further, a variety of related studies have found that the basal ganglia is sensitive to syntactic complexity (Prat and Just, 2011) and syntactic anomaly (Moro et al., 2001). Thus, this result is consistent with the region being engaged in the network that computes syntax.

### Interaction effect in MFG/SFG

The effect of syntactic size demonstrated an effect beyond type-identity interveners, bilaterally in the MFG/SFG, though most predominantly in the SFG. This was an unexpected finding, particularly with respect to the peak activation that lies anteriorly. The more posterior extent of the activation observed in the left hemisphere is similar to that seen in studies investigating the processing of Japanese scrambled sentences (Kinno et al., 2008) and one study that was interested in general distance effects within subject-verb agreement dependencies (Makuuchi et al., 2009). In fact, this posterior area is in very close proximity to the posterior end of the linear effect cluster (that is extending beyond Broca's area). Thus, this area seems to demonstrate some general engagement when Working Memory increases during sentence processing.

The bulk of the activation for the interaction, however, extends further anteriorly and is bilateral, thus demonstrating a distinction from these previous studies. Interpretations of the effect should, therefore, be made cautiously. Moreover, the plot of percent signal change by condition within this cluster provides a difficult picture to interpret. It seems as though there is activation for 1 intervening object in the double object construction, but no activation with 1 intervening object in the embedded clause structure. Then the pattern inverts for 2 intervening objects.

### Auxiliary brain areas and contrasts

#### Superior temporal cortex and CP>NP

In addition to examining the effect of distance (similarity and syntactic size) we looked at differences between the two Types of constructions. The results of this contrast need to be treated with care since many syntactic variables are concurrently manipulated (since this was not our primary interest). Although the CP and NP conditions contain the same number of NPs, the CP condition has an additional verb, whereas the NP condition has the preposition *to*. Furthermore, the argument structure of the verbs differ, the NP condition contains ditransitive verbs that the CP condition does not. Rather the CP condition contains more verbs that take sentential complements. Given these differences in verb argument structure, there is a consequent effect on the degree of syntactic structure building. The CP condition embeds clauses on each verb, thereby generating more syntactic structure. Having acknowledged the variety of differences across the TYPE contrast, the results of this contrast can be used to speak to current functional interpretations of the regions observed by this contrast that make reference to these syntactic variables. The anterior-to-posterior superior temporal gyrus activation is consistent with previous studies that have found the mid-to-superior posterior temporal gyrus sensitive to argument structure and syntax (Ben-Shachar et al., 2003, 2004; Friederici et al., 2009; Santi and Grodzinsky, 2010) and the anterior temporal cortex to structure building (Humphries et al., 2005; Rogalsky and Hickok, 2009; Brennan et al., 2012). It is of interest to note, however, that the peak and focus of the activation is in the middle temporal cortex and not more anteriorly. In fact, in lowering the *p*-value (*p* < 0.001), the anterior temporal activation disappears and the mid-to-posterior activation remains. Thus, this contrast predominately depends on the mid-to-posterior superior temporal gyrus, rather than its anterior portion.

In looking at the percent signal change across conditions for this cluster, it is clear this effect is observed regardless of the number of intervening NPs. Additionally, it suggests a linear trend in activation with increasing number of NP interveners for the condition with multiply embedded CPs that is not observed for the condition with a ditransitive verb in a singly embedded CP. Similarly, the offline behavioral RT data demonstrate some evidence for increasing RTs with increasing number of interveners in the multiply embedded clause condition. This was observed in a marginally significant interaction effect (only in the by-subjects analysis, however). At appropriately thresholded levels, the fMRI data do not demonstrate such an interaction in the STS. However, when the voxel-wise *p*-value is dropped to *p* < 0.05 (voxel-wise and uncorrected for cluster size) the STS is observed in the interaction effect map. That is, a greater linear increase in activation with an increasing number of intervening NPs is observed in the multiply embedded clause condition over the single embedded clause one. Recall, in the two intervening object NP condition, the movement is occurring over a clausal boundary in the multiple clause condition, but is within a clause in the double object condition. Wagers and Phillips (2014) demonstrate that not all properties of the filler are maintained across a clause boundary, requiring their retrieval at the gap site. Thus the data, though not significant, show a trend for the STS to be sensitive to multiple movements or retrieval demands.

#### Inferior occipital gyrus and CP>NP

Not only was the superior temporal sulcus activated by the contrast in syntax type, but so was the inferior occipital gyrus. The location of activation is consistent with that observed by Makuuchi et al. (2013) in their contrast between Topicalization and Scrambling. The authors interpret this to be due to visual attention driven by the case-marked NP that appears sentence initial in wh-movement (topicalization), but in the embedded clause in scrambled structures. If the activation is due to increased attention, then in this study it must be for a different reason than the presence of an early case-marked NP that predicts the additional NPs. First there is no case-marking in the present study and, if anything, the filler appears earlier in the NP condition than the CP condition. A distinct potential syntactic factor that could be generating predictions and increasing visual attention in the CP compared to NP condition is that there are more open clausal phrases in the CP condition, leading to more predictions of verbs and arguments. Generally, these findings are consistent with other studies that demonstrated top-down effects in visual areas based on lexical predictions (Dikker and Pylkkänen, 2011).

## Conclusions

The current parametric study manipulated the number of NP interveners in a movement dependency while also manipulating the presence of a clausal boundary across such a dependency. The results demonstrated a linear effect of number of interveners in Broca's area but no interaction between the number of interveners and the presence of a clausal boundary. More superiorly and bilaterally in the SFG there was an interaction due to the clausal boundary having a greater effect on the number of interveners than NPs. The STS demonstrated greater activation for the multiple clausal embedding condition than the single clausal embedding condition regardless of the number of interveners. As there were multiple distinctions across these conditions it is difficult to attribute the activation to a particular factor. Further, there was a trend within the STS in being sensitive to movement over a clause boundary, although not significant. In conclusion, Broca's area is sensitive to the number of interveners that are similar to the moved constituent and the activation is not augmented by an additional movement, or movement over a clausal boundary. Thus, type-identical interference rather than movement or crossing of a clausal boundary increases activation in Broca's area.

### Conflict of interest statement

The authors declare that the research was conducted in the absence of any commercial or financial relationships that could be construed as a potential conflict of interest.

## Footnotes

^1^Although note that in (7) the NP is modified by the adjective, “dangerous,” whereas in (8) it is not. Thus type similarity between the intervening NP and the head of the dependency also differs across this contrast.

^2^This is relevant given that Glaser et al. (2013) compared an intervening CP to an intervening PP in testing effects of (subject) NP interference, where the intervening CP condition was also the condition with “high-syntactic interference” and resulted in greater activation in Broca's area. The question remains whether this greater activation is due to greater syntactic structure intervening the dependency or the subject status of the NP within this structure.

^3^This level of accuracy is based on the fact that the sentences are quite complicated and additionally the offline comprehension questions were difficult, as they involved a thematic role reversal. Further, it is not necessarily the case that incorrect answers to an off-line comprehension question correspond to an incorrect parse online. Rather it may simply be the product of an incorrect memory of that parse. As will be discussed later on, in the lab prior to fMRI scanning, participants performed at 75% or higher in each condition and as is normal, performance became a bit worse in the peculiar environment of an fMRI machine. Only two subjects had an individual condition with an average accuracy of 65%, but over all conditions each subject performed above 70% and typically above 80%.

^4^An alternative baseline with no movement, but rather a complementizer was considered. It was ruled out as problematic, given it would be the only condition without a wh-phrase. This confound would make interpretations of the data difficult.
