# Supplementary material for: An fMRI study dissociating distance measures computed by Broca's area in movement processing: clause boundary vs. identity
Source: Front Psychol. 2015 May 20;6:654. doi: 10.3389/fpsyg.2015.00654 (PMC4438592; doi:10.3389/fpsyg.2015.00654)
Supplement: Supplementary file 1 [file Table1.PDF]

Condition	Item	Sentence
CPS0	1	I said the neurosurgeon knew which resident liked the porter
	2	She said the ambassador announced which lawyer fired the salesman
	3	He said the grumpy baker learned which florist hugged the prime minister
	4	We said the informant knew which officer hit the cab driver
	5	I said the house cleaner announced which mom kissed the friendly neighbour
	6	She said the victim learned which counselor forgave the skeptical juror
	7	He said the repairman knew which consumer answered the overseer
	8	We said the waitress announced which mafia boss hired the loan shark
	9	I said the son learned which teenager ignored the babysitter
	10	She said the administrator knew which plaintiff sued the producer
	11	I thought the detective knew which bouncer tripped the paparazzi
	12	She thought the boss announced which programmer employed the IT guy
	13	He thought the investor learned which employee praised the civil servant
	14	We thought the agent knew which editor blackmailed the publisher
	15	I thought the cellist announced which viewer assisted the violinist
	16	She thought the manager learned which solicitor served the witness
	17	He thought the volunteer knew which chemist married the pharmacist
	18	We thought the banker announced which mobster threatened the accountant
	19	I thought the surveyor learned which mapmaker paid the carpenter
	20	She thought the librarian knew which athletic coach noticed the blond girl
	21	I claimed the waiter knew which celebrity loved the disc-jockey
	22	She claimed the tired medic announced which patient attacked the doctor
	23	He claimed the supervisor learned which prisoner kidnapped the judge
	24	We claimed the old care-giver knew which shy boy approached the play-mate
	25	I claimed the sales-rep announced which customer phoned the supplier
	26	She claimed the security guard learned which visitor abused the king
	27	He claimed the politician knew which mayor defended the president
	28	We claimed the nice old lady announced which housewife warned the renters
	29	I claimed the bartender learned which obnoxious girl recognized the busboy
	30	She claimed the lab assistant knew which scientist watched the garbage man
	31	I hoped the curator knew which gardener avoided the therapist
	32	She hoped the veterinarian announced which breeder confused the client
	33	He hoped the actor learned which singer protected the PR consultant
	34	We hoped the planner knew which developer chose the electrician
	35	I hoped the dealer announced which poker player scammed the high roller
	36	She hoped the coordinator learned which bride grabbed the photographer
	37	He hoped the clerk knew which recruiter insulted the executive
	38	We hoped the real-estate agent announced which notary billed the buyer
	39	I hoped the negotiator learned which translator followed the indian
	40	She hoped the newscaster knew which actress dated the interviewee
CPO1	1	I said the neurosurgeon knew which porter the resident liked
	2	She said the ambassador announced which salesman the lawyer fired
	3	He said the grumpy baker learned which prime minister the florist hugged
	4	We said the informant knew which cab driver the officer hit
	5	I said the house cleaner announced which friendly neighbour the mom kissed
	6	She said the victim learned which skeptical juror the counselor forgave
	7	He said the repairman knew which overseer the consumer answered
	8	We said the waitress announced which loan shark the mafia boss hired
	9	I said the son learned which babysitter the teenager ignored
	10	She said the administrator knew which producer the plaintiff sued
	11	I thought the detective knew which paparazzi the bouncer tripped
	12	She thought the boss announced which IT guy the programmer employed
	13	He thought the investor learned which civil servant the employee praised
	14	We thought the agent knew which publisher the editor blackmailed
	15	I thought the cellist announced which violinist the viewer assisted
	16	She thought the manager learned which witness the solicitor served
	17	He thought the volunteer knew which pharmacist the chemist married
	18	We thought the banker announced which accountant the mobster threatened
	19	I thought the surveyor learned which carpenter the mapmaker paid
	20	She thought the librarian knew which blond girl the athletic coach noticed
	21	I claimed the waiter knew which disc-jockey the celebrity loved
	22	She claimed the tired medic announced which doctor the patient attacked
	23	He claimed the supervisor learned which judge the prisoner kidnapped
	24	We claimed the old care-giver knew which play-mate the shy boy approached

	25	I claimed the sales-rep announced which supplier the customer phoned
	26	She claimed the security guard learned which king the visitor abused
	27	He claimed the politician knew which president the mayor defended
	28	We claimed the nice old lady announced which renters the housewife warned
	29	I claimed the bartender learned which busboy the obnoxious girl recognized
	30	She claimed the lab assistant knew which garbage man the scientist watched
	31	I hoped the curator knew which therapist the gardener avoided
	32	She hoped the veterinarian announced which client the breeder confused
	33	He hoped the actor learned which PR consultant the singer protected
	34	We hoped the planner knew which electrician the developer chose
	35	I hoped the dealer announced which high roller the poker player scammed
	36	She hoped the coordinator learned which photographer the bride grabbed
	37	He hoped the clerk knew which executive the recruiter insulted
	38	We hoped the real-estate agent announced which buyer the notary billed
	39	I hoped the negotiator learned which indian the translator followed
	40	She hoped the newscaster knew which interviewee the actress dated
CPO2	1	I knew which porter the neurosurgeon said the resident liked
	2	She announced which salesman the ambassador said the lawyer fired
	3	He learned which prime minister the grumpy baker said the florist hugged
	4	We knew which cab driver the informant said the officer hit
	5	I announced which friendly neighbour the house cleaner said the mom kissed
	6	She learned which skeptical juror the victim said the counselor forgave
	7	He knew which overseer the repairman said the consumer answered
	8	We announced which loan shark the waitress said the mafia boss hired
	9	I learned which babysitter the son said the teenager ignored
	10	She knew which producer the administrator said the plaintiff sued
	11	I knew which paparazzi the detective thought the bouncer tripped
	12	She announced which IT guy the boss thought the programmer employed
	13	He learned which civil servant the investor thought the employee praised
	14	We knew which publisher the agent thought the editor blackmailed
	15	I announced which violinist the cellist thought the viewer assisted
	16	She learned which witness the manager thought the solicitor served
	17	He knew which pharmacist the volunteer thought the chemist married
	18	We announced which accountant the banker thought the mobster threatened
	19	I learned which carpenter the surveyor thought the mapmaker paid
	20	She knew which blond girl the librarian thought the athletic coach noticed
	21	I knew which disc-jockey the waiter claimed the celebrity loved
	22	She announced which doctor the tired medic claimed the patient attacked
	23	He learned which judge the supervisor claimed the prisoner kidnapped
	24	We knew which play-mate the old care-giver claimed the shy boy approached
	25	I announced which supplier the sales-rep claimed the customer phoned
	26	She learned which king the security guard claimed the visitor abused
	27	He knew which president the politician claimed the mayor defended
	28	We announced which renters the nice old lady claimed the housewife warned
	29	I learned which busboy the bartender claimed the obnoxious girl recognized
	30	She knew which garbage man the lab assistant claimed the scientist watched
	31	I knew which therapist the curator hoped the gardener avoided
	32	She announced which client the veterinarian hoped the breeder confused
	33	He learned which PR consultant the actor hoped the singer protected
	34	We knew which electrician the planner hoped the developer chose
	35	I announced which high roller the dealer hoped the poker player scammed
	36	She learned which photographer the coordinator hoped the bride grabbed
	37	He knew which executive the clerk hoped the recruiter insulted
	38	We announced which buyer the real-estate agent hoped the notary billed
	39	I learned which indian the negotiator hoped the translator followed
	40	She knew which interviewee the newscaster hoped the actress dated
NPS0	1	I knew which veterinarian introduced the breeder to the client
	2	She announced which ambassador introduced the lawyer to the salesman
	3	He learned which newscaster introduced the actress to the interviewee
	4	We knew which informant introduced the officer to the cab driver
	5	I announced which house cleaner introduced the mom to the friendly neighbour
	6	She learned which coordinator introduced the bride to the photographer
	7	He knew which sales-rep introduced the customer to the supplier
	8	We announced which agent introduced the editor to the publisher
	9	I learned which son introduced the teenager to the babysitter

	10	She knew which waitress introduced the mafia boss to the loan shark
	11	I knew which tired medic recommended the doctor to the patient
	12	She announced which boss recommended the programmer to the IT guy
	13	He learned which supervisor recommended the judge to the prisoner
	14	We knew which actor recommended the PR consultant to the singer
	15	I announced which cellist recommended the violinist to the viewer
	16	She learned which manager recommended the solicitor to the witness
	17	He knew which volunteer recommended the chemist to the pharmacist
	18	We announced which banker recommended the accountant to the mobster
	19	I learned which surveyor recommended the mapmaker to the carpenter
	20	She knew which old care-giver recommended the play-mate to the shy boy
	21	I knew which waiter described the celebrity to the disc-jockey
	22	She announced which investor described the employee to the civil servant
	23	He learned which detective described the bouncer to the paparazzi
	24	We knew which librarian described the athletic coach to the blond girl
	25	I announced which administrator described the plaintiff to the clerk
	26	She learned which security guard described the visitor to the king
	27	He knew which politician described the mayor to the president
	28	We announced which nice old lady described the housewife to the renters
	29	I learned which bartender described the obnoxious girl to the busboy
	30	She knew which lab assistant described the scientist to the garbage man
	31	I knew which real-estate agent showed the notary to the buyer
	32	She announced which neurosurgeon showed the resident to the porter
	33	He learned which repairman showed the consumer to the overseer
	34	We knew which curator showed the gardener to the electrician
	35	I announced which planner showed the developer to the therapist
	36	She learned which victim showed the counselor to the skeptical juror
	37	He knew which producer showed the recruiter to the executive
	38	We announced which dealer showed the poker player to the high roller
	39	I learned which negotiator showed the translator to the indian
	40	She knew which grumpy baker showed the florist to the prime minister
NPO1	1	I knew which breeder the veterinarian introduced to the client
	2	She announced which lawyer the ambassador introduced to the salesman
	3	He learned which actress the newscaster introduced to the interviewee
	4	We knew which officer the informant introduced to the cab driver
	5	I announced which mom the house cleaner introduced to the friendly neighbour
	6	She learned which bride the coordinator introduced to the photographer
	7	He knew which customer the sales-rep introduced to the supplier
	8	We announced which editor the agent introduced to the publisher
	9	I learned which teenager the son introduced to the babysitter
	10	She knew which mafia boss the waitress introduced to the loan shark
	11	I knew which doctor the tired medic recommended to the patient
	12	She announced which programmer the boss recommended to the IT guy
	13	He learned which judge the supervisor recommended to the prisoner
	14	We knew which PR consultant the actor recommended to the singer
	15	I announced which violinist the cellist recommended to the viewer
	16	She learned which solicitor the manager recommended to the witness
	17	He knew which chemist the volunteer recommended to the pharmacist
	18	We announced which accountant the banker recommended to the mobster
	19	I learned which mapmaker the surveyor recommended to the carpenter
	20	She knew which play-mate the old care-giver recommended to the shy boy
	21	I knew which celebrity the waiter described to the disc-jockey
	22	She announced which employee the investor described to the civil servant
	23	He learned which bouncer the detective described to the paparazzi
	24	We knew which athletic coach the librarian described to the blond girl
	25	I announced which plaintiff the administrator described to the clerk
	26	She learned which visitor the security guard described to the king
	27	He knew which mayor the politician described to the president
	28	We announced which housewife the nice old lady described to the renters
	29	I learned which obnoxious girl the bartender described to the busboy
	30	She knew which scientist the lab assistant described to the garbage man
	31	I knew which notary the real-estate agent showed to the buyer
	32	She announced which resident the neurosurgeon showed to the porter
	33	He learned which consumer the repairman showed to the overseer
	34	We knew which gardener the curator showed to the electrician

	35	I announced which developer the planner showed to the therapist
	36	She learned which counselor the victim showed to the skeptical juror
	37	He knew which recruiter the producer showed to the executive
	38	We announced which poker player the dealer showed to the high roller
	39	I learned which translator the negotiator showed to the indian
	40	She knew which florist the grumpy baker showed to the prime minister
NPO2	1	I knew which client the veterinarian introduced the breeder to
	2	She announced which salesman the ambassador introduced the lawyer to
	3	He learned which interviewee the newscaster introduced the actress to
	4	We knew which cab driver the informant introduced the officer to
	5	I announced which friendly neighbour the house cleaner introduced the mom to
	6	She learned which photographer the coordinator introduced the bride to
	7	He knew which supplier the sales-rep introduced the customer to
	8	We announced which publisher the agent introduced the editor to
	9	I learned which babysitter the son introduced the teenager to
	10	She knew which loan shark the waitress introduced the mafia boss to
	11	I knew which patient the tired medic recommended the doctor to
	12	She announced which IT guy the boss recommended the programmer to
	13	He learned which prisoner the supervisor recommended the judge to
	14	We knew which singer the actor recommended the PR consultant to
	15	I announced which viewer the cellist recommended the violinist to
	16	She learned which witness the manager recommended the solicitor to
	17	He knew which pharmacist the volunteer recommended the chemist to
	18	We announced which mobster the banker recommended the accountant to
	19	I learned which carpenter the surveyor recommended the mapmaker to
	20	She knew which shy boy the old care-giver recommended the play-mate to
	21	I knew which disc-jockey the waiter described the celebrity to
	22	She announced which civil servant the investor described the employee to
	23	He learned which paparazzi the detective described the bouncer to
	24	We knew which blond girl the librarian described the athletic coach to
	25	I announced which clerk the administrator described the plaintiff to
	26	She learned which king the security guard described the visitor to
	27	He knew which president the politician described the mayor to
	28	We announced which renters the nice old lady described the housewife to
	29	I learned which busboy the bartender described the obnoxious girl to
	30	She knew which garbage man the lab assistant described the scientist to
	31	I knew which buyer the real-estate agent showed the notary to
	32	She announced which porter the neurosurgeon showed the resident to
	33	He learned which overseer the repairman showed the consumer to
	34	We knew which electrician the curator showed the gardener to
	35	I announced which therapist the planner showed the developer to
	36	She learned which skeptical juror the victim showed the counselor to
	37	He knew which executive the producer showed the recruiter to
	38	We announced which high roller the dealer showed the poker player to
	39	I learned which indian the negotiator showed the translator to
	40	She knew which prime minister the grumpy baker showed the florist to
