# Supplementary figures and images for: An fMRI study dissociating distance measures computed by Broca's area in movement processing: clause boundary vs. identity
Source: Front Psychol. 2015 May 20;6:654. doi: 10.3389/fpsyg.2015.00654 (PMC4438592; doi:10.3389/fpsyg.2015.00654)

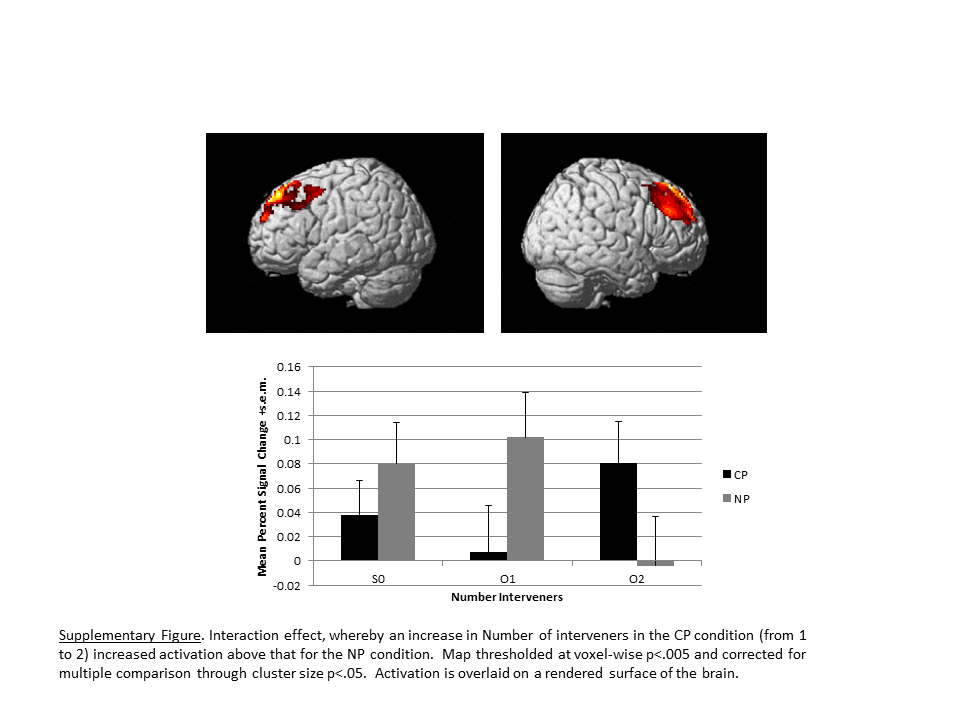

Supplement: Supplementary file 2 [file Image1.TIF]
